# Deep Learning-Based State-of-Health Estimation of Proton-Exchange Membrane Fuel Cells under Dynamic Operation Conditions

**DOI:** 10.3390/s24144451

**Published:** 2024-07-10

**Authors:** Yujia Zhang, Xingwang Tang, Sichuan Xu, Chuanyu Sun

**Affiliations:** 1 School of Automotive Studies, Tongji University, Shanghai 201804, China; 2 School of Electrical Engineering and Automation, Harbin Institute of Technology, Harbin 150001, China

**Keywords:** PEMFC, dynamic load cycle test, deep learning model, state-of-health estimation, degradation prediction

## Abstract

Proton-exchange membrane fuel cells (PEMFCs) play a crucial role in the transition to sustainable energy systems. Accurately estimating the state of health (SOH) of PEMFCs under dynamic operating conditions is essential for ensuring their reliability and longevity. This study designed dynamic operating conditions for fuel cells and conducted durability tests using both crack-free fuel cells and fuel cells with uniform cracks. Utilizing deep learning methods, we estimated the SOH of PEMFCs under dynamic operating conditions and investigated the performance of long short-term memory networks (LSTM), gated recurrent units (GRU), temporal convolutional networks (TCN), and transformer models for SOH estimation tasks. We also explored the impact of different sampling intervals and training set proportions on the predictive performance of these models. The results indicated that shorter sampling intervals and higher training set proportions significantly improve prediction accuracy. The study also highlighted the challenges posed by the presence of cracks. Cracks cause more frequent and intense voltage fluctuations, making it more difficult for the models to accurately capture the dynamic behavior of PEMFCs, thereby increasing prediction errors. However, under crack-free conditions, due to more stable voltage output, all models showed improved predictive performance. Finally, this study underscores the effectiveness of deep learning models in estimating the SOH of PEMFCs and provides insights into optimizing sampling and training strategies to enhance prediction accuracy. The findings make a significant contribution to the development of more reliable and efficient PEMFC systems for sustainable energy applications.

## 1. Introduction

Green and low-carbon development has become the overarching trend in global energy evolution, with hydrogen energy emerging as the most promising secondary green energy source. Compared to the intermittent and seasonal variations in solar and wind energy, hydrogen energy can be stored on a large scale for a long time and provide a stable and sustainable energy supply [1,2,3]. PEMFCs play a pivotal role in this landscape as the key energy conversion devices within the hydrogen industry chain [4,5]. Their inherent advantages—zero carbon emissions, the ability to start in low temperatures, high power density, and low noise levels—position them as critical components in the advancement of various sectors, including transportation, industry, construction, and power generation [6]. However, the operational conditions encountered in practical applications are often complex and highly variable. Particularly in vehicles, the dynamic changes in driving conditions—such as variations in speed, load, and environmental factors—can lead to catalyst loss and agglomeration, as well as structural damage to the membrane electrode assembly [7,8]. These issues ultimately result in performance degradation and reduced durability of the fuel cells. This degradation is influenced by multiple factors, including temperature fluctuations, humidity, load cycles, and impurities in the hydrogen supply.

To address these challenges, prognostic and health management (PHM) technology has been developed. PHM technology is designed to ensure the long-term serviceability of PEMFCs by providing a systematic approach to monitoring, diagnosing, and predicting the health of the cells [9]. The core concept of PHM involves using historical data and real-time operating information to estimate the current SOH of PEMFCs [10]. In order to predict the long-term degradation of PEMFCs, Lv et al. [11] applies a transformer-based PEMFC prognostic framework, Zhang et al. [12] chooses a long short-term memory model, Wang et al. [13] deploys a gated recurrent unit, and Pan et al. [14] uses a temporal convolutional network to predict the future performance trends of PEMFCs. By understanding the degradation mechanisms and predicting potential failures, maintenance can be scheduled proactively based on the actual operating condition of the fuel cells, thereby enhancing the durability of the fuel cell system [15].

Estimating the SOH of the PEMFCs involves three advanced methods, each with its own advantages and applications: model-based methods, data-driven methods, and hybrid methods [16].

Model-based methods rely on physical or chemical models to describe the internal processes and behaviors of fuel cells. These methods utilize mathematical equations and simulations to predict the SOH based on known internal characteristics and reactions. The advantage of model-based methods lies in their ability to provide a deep understanding of the fundamental mechanisms driving fuel cell performance and degradation. However, they require precise knowledge of the PEMFC system and can be complex to develop and implement [17]. Wang et al. [18] introduced a polarization resistance to indicate the degradation of PEMFCs. Ou et al. [19] established a voltage degradation model integrating electrochemical surface area (ECSA) and equivalent resistance, and introduced a recovery factor to characterize the voltage recovery phenomenon of PEMFCs after shutdown and restart. Pei et al. [20] predicted the lifespan of fuel cells using the current degradation law through a first-order kinetic model, introducing a scale factor *k* to account for degradation factors. Yang et al. [21] presented Bayesian framework-based model uncertainty and state simultaneous estimation (Bayes-MUSE) method for robust aging prediction and remaining-useful-life estimation. Zhao et al. [22] characterized degradation using parameters like hydrogen crossover, membrane resistance increase, and ECSA loss, aligning well with polarization curves.

In contrast, data-driven methods utilize statistical and machine learning techniques to analyze historical and real-time data collected from fuel cells. By identifying patterns and correlations within the data, these methods predict the SOH without detailed knowledge of underlying physical processes. Data-driven methods are more flexible and easier to implement when dealing with complex and variable operating conditions [23]. Particularly in practical applications, where conditions are complex and variable, data-driven methods can capture this complexity by analyzing large amounts of data [24]. Due to the close correlation between the degradation process of fuel cells and operational time, recurrent neural networks (RNNs) and their variants and convolutional neural network (CNN) architecture, which excel in predicting time-series data, have been selected and validated in many studies. Sun et al. [25] used complete ensemble empirical mode decomposition to analyze voltage sequences, which were then fed into CNN–LSTM models for accurate prediction. Yue et al. [26] employed a conditional CNN to extract features and a recursive strategy for iterative future predictions. Teng et al. [27] applied Holt–Winters smoothing to enhance PEMFC degradation features and used a multivariate CNN for multi-step prediction with limited data. He et al. [28] used an auto-encoder for health indicators and LSTM for precise prognosis, showing better predictions than other methods across different loads. In addition, transformer-based models are also very good at predicting temporal data tasks. Fu et al. [29] used a novel non-stationary transformer model and Zhou et al. [30] added a convolutional network to the transformer. They applied transformer to the field of fuel cell lifetime prediction and improved the prediction accuracy through different methods.

Hybrid methods combine elements of both model-based and data-driven approaches to leverage the strengths of each. These methods use physical models to provide a foundational understanding of fuel cell systems and enhance the accuracy of data-driven predictions. By integrating the two approaches, hybrid methods offer a more comprehensive and reliable estimation of the SOH of PEMFCs. They can compensate for limitations of each individual method, providing more accurate and reliable diagnostics and prognostics. Hong et al. [31] obtained voltage output with multiple stochastic conditions imported based on a semi-empirical model, which was selected as a health indicator characterizing the SOH for the fuel cells and entered into the LSTM model for training. Wang et al. [32] developed a hybrid model that used the results of prediction results of the semi-empirical model to modify the input of the data-driven model. It was found that the semi-empirical degradation model can effectively improve the oscillation of prediction results of the data-driven model during long-term degradation. However, hybrid methods also have limitations. Firstly, they may require more time and resources due to the development and maintenance of physical models and data collection and processing. Secondly, integrating models and data may increase complexity, making implementation and interpretation more challenging. Additionally, the performance of hybrid methods heavily relies on the effective integration of models and data, and improper integration may lead to estimation biases and errors.

Currently, with the expansion of application domains such as the automotive industry, the operational load conditions have become increasingly complex, highlighting the necessity for research on PEMFCs under dynamic load conditions [33]. However, there is a scarcity of studies on dynamic load durability tests in the existing literature. Consequently, conducting dynamic load durability testing and developing predictive models based on these tests are both crucial and significant. In light of this, this paper undertook 550 h durability experiments on two fuel cells and carried out a comparative analysis of several deep learning methods using dynamic load condition test data. The principal contributions of this paper are summarized as follows.

1. A dynamic load cycle durability test for PEMFCs was developed. The experimental procedure included designing a suitable dynamic load cycle, determining the optimal operating parameters, and performing dynamic load durability testing on two fuel cells. The durability tests lasted for over 550 h, with all data being collected using a G20 test station.
2. Four deep learning models were selected to perform the prediction tasks. These models were LSTM, GRU, TCN, and transformer, and were utilized to predict the degradation of the fuel cells. The predictive performance of these different models was thoroughly evaluated.
3. The impact of durability data resampling intervals and model training data proportions on prediction accuracy was investigated. This study was validated and compared using two durability datasets.

The reminder of this paper is organized as follows. The process of preparing two fuel cells and the dynamic load cycle durability experiment are introduced in Section 2. The main methods used in the subsequent section, such as preliminary work on data processing and the principles of neural networks for prediction, are discussed in Section 3. Section 4 describes the prediction results, followed by an interpretation and analysis of the results. Finally, the conclusions are presented in Section 5.

## 2. Experimental

### 2.1. Preparation of Membrane Electrode Assembly

A well-constructed membrane electrode assembly, the most fundamental component in PEMFCs, is essential for achieving high-performance and reliable fuel cells. In this paper, the proton-exchange membrane is GORE-SELECT^®^ M788.12, while the 46.5 wt% Pt/C catalyst is produced by TANAKA and the gas diffusion layer is SGL Sigracet 22 BB. The catalyst layer was prepared using the ultrasonic spray-coating method, where the catalyst ink was directly sprayed onto the proton-exchange membrane to prepare the catalyst-coated membrane. The basic technical parameters of the two fuel cells are listed in Table 1.

The interactions between catalysts, ionomers, and solvents in the ink system play a significant role in determining the final structure of membrane electrode assembly [34]. How to choose solvents significantly impacts the dispersion behavior of polymer clusters within the ink. In this research, isopropanol, ethanol, and pure water were meticulously selected for the preparation of the catalyst ink. The precise composition ratios were 46 wt% isopropanol, 46 wt% ethanol, and 46 wt% pure water. Additionally, ionomers not only serve as proton transport channels within the catalyst layer but also enhance the stability of the catalyst ink [35,36]. However, an excessive quantity of ionomers can obstruct the pores of the catalyst layer, leading to reduced mass transfer capabilities. Therefore, the mass ratio of dehydrated ionomers to carbon support is set to 0.6.

**Figure 1:**
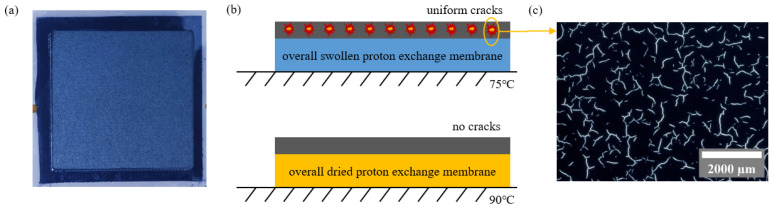
Schematic diagram of the prepared membrane electrode assembly. (**a**) Membrane electrode assembly; (**b**) membrane electrode assembly with uniform cracks and no cracks; (**c**) cracks under a scanning electron microscope.

During the spraying process, when the temperature of the vacuum adsorption table is relatively low, the internal stress accumulated within the catalyst layer during the drying process will be greater than the internal stress caused by the rapid evaporation of the ink, ultimately leading to tearing and fracturing of the catalyst layer at its weakest point in the porous structure. Uniform swelling occurs throughout the catalyst layer at 75 °C, resulting in relatively uniform cracks, which can be observed under a scanning electron microscope, as shown in Figure 1. When the temperature reaches 90 °C, the ink quickly evaporates upon contact with the catalyst layer, effectively reducing the magnitude of internal stress during the drying process, preventing crack formation in the catalyst layer.

### 2.2. PEMFC Dynamic Durability Test

**Figure 2:**
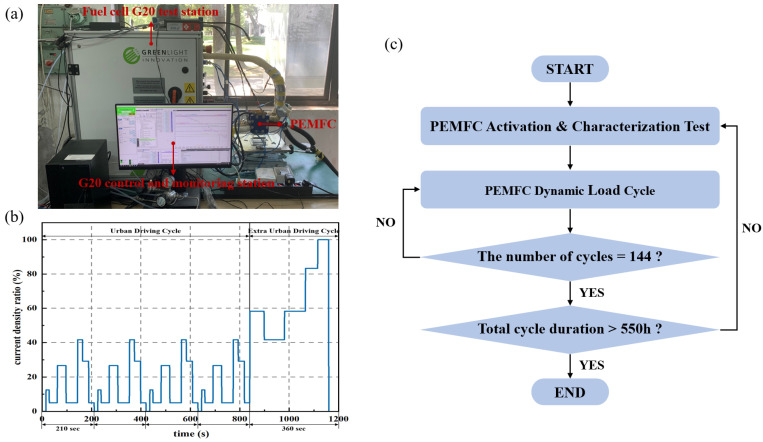
G20 test station and schematic diagram of the dynamic load durability cycle and test protocol. (**a**) Fuel cell G20 test station; (**b**) dynamic load durability cycle; (**c**) test protocol.

The durability cycle test employed in this study is derived from the New European Driving Cycle. This methodology, as proposed by Tsotridis et al. [37], converts the speed-versus-time relationship of the original cycle into a current density ratio-versus-time relationship, thus producing a dynamic load cycle for the fuel cell (FC-DLC). We added sections with a current density of zero to the original FC-DLC to simulate the idling conditions that may occur during actual driving, and the improved dynamic load durability cycle is shown in Figure 2b. Each cycle comprises 35 test steps for a total duration of 1200 s. To conduct the fuel cell dynamic load cycle on the G20 test bench, the rated operating point of the PEMFC is defined by the operating state at which the fuel cell output voltage is 0.65 V [38] and relative humidity is 100%. The current value of the PEMFC at this rated operating state corresponds to 100% load in our profile. It is worth noting that different PEMFCs have different rated operating points, and this point needs to be determined before the durability test begins. In this article, the rated current density of the crack-free PEMFC is 500 mA/cm^2^, while the rated working current density of the PEMFC with uniform cracks is 520 mA/cm^2^.

After assembling the fuel cell on the G20 test bench, the operating conditions were set to an operating temperature of 80 °C, reaction gas relative humidity of 100%, and inlet pressure of anode and cathode of 100 kPa to begin the durability test procedure shown in Figure 2. The anode and cathode gases are hydrogen and air, respectively. Before the formal durability testing, an activation process is performed, and electrochemical methods are used to characterize the performance of the fuel cell to ensure stable and reliable installation. The purpose of the characterization test is to record the polarization curves of PEMFCs. A test period is set to 144 FC-DLC cycles (approximately 50 h). After completing one test period, the dynamic load cycling test is stopped, and the fuel cell undergoes another activation and characterization test. The overall durability testing lasts for approximately 550 h.

## 3. Materials and Methods

**Figure 3:**
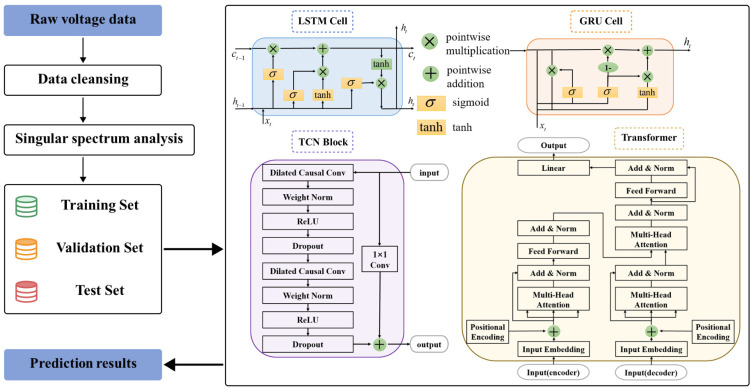
The flowchart and structural diagram of the model used in this paper.

In this section, the method of handling data and the deep learning model used in this paper are introduced. Figure 3 shows the flowchart and schematic diagrams of the model structures.

### 3.1. Singular Spectrum Analysis

Singular spectrum analysis (SSA) [39] decomposes and reconstructs the trajectory matrix of the time series under study to extract different component series, such as long-term trends, seasonal trends, and noise, which reveals the remarkable dynamics [40] in nonlinear and nonstationary time series. The detailed steps are as follows.

Firstly, SSA transforms the given time series $\left\{x_{1},x_{2},\cdots ,x_{N}\right\}$ into a trajectory matrix *X* with an embedding dimension *L*. Each column of the trajectory matrix *X* is a length-*L* subsequence of the time series:

(1) $$X=\left(\begin{array}{cccc} x_{1} & x_{2} & \cdots & x_{K}\\ x_{2} & x_{3} & \cdots & x_{K+1}\\ \vdots & \vdots & \ddots & \vdots \\ x_{L} & x_{L+1} & \cdots & x_{N} \end{array}\right)$$

where *L* is the window length, *N* is the length of the time series, and K=N−L+1.

Second, this matrix undergoes singular value decomposition to extract its singular values and vectors by $X=U\sum V^{T}$, where U is an L×L orthogonal matrix containing the left singular vectors, ∑ is an L×K diagonal matrix containing the singular values, and V is an L×K orthogonal matrix containing the right singular vectors.

Third, the leading singular values and vectors are selected to reconstruct the trajectory matrix. The matrix ∑ is decomposed into r submatrices, obtaining $X_{i}=\sigma_{i}u_{i}v_{i}^{T}$, where $\sigma_{i}$ is the *i*th singular value and $u_{i}$ and $v_{i}$ are the corresponding left and right singular vectors. An appropriate r needs to be chosen to reconstruct $X=X_{1}+X_{2}+\cdots +X_{r}$.

Finally, apply diagonal averaging to the reconstructed matrix to obtain the new time series by Equation (2):

(2) $$y_{k}=\frac{1}{k}\sum_{i+j=k+1}x_{ij}$$

where $y_{k}$ is the reconstructed value of the time series.

### 3.2. Long Short-Term Memory

LSTM is a type of recurrent neural network (RNN) architecture designed to capture long-term dependencies in sequential data [41]. Unlike traditional RNNs, LSTM introduces gating mechanisms to retain or discard specific features of the data, effectively addressing the vanishing and exploding gradient problems. This enables LSTM networks to learn and remember information over longer periods, making them well suited for tasks involving sequential or time-series data [42].

An LSTM memory cell controls the flow of information through three gates. The “forget” gate determines which information in the memory cell needs to be discarded. Its output value ranges from 0 to 1, with 0 indicating complete discarding and 1 indicating complete retention. The input gate decides which new information needs to be stored in the memory cell. The output gate determines which information from the memory cell needs to be output to the next time step. The inputs to these gates include the current time step’s input and the previous time step’s hidden state. The formulas for calculating the outputs are as follows:

(3) $$f_{t}=\sigma (W_{f}\cdot [h_{t-1},x_{t}]+b_{f})$$

(4) $$i_{t}=\sigma (W_{i}\cdot [h_{t-1},x_{t}]+b_{i})$$

(5) $$o_{t}=\sigma (W_{o}\cdot [h_{t-1},x_{t}]+b_{o})$$

where $W_{f},W_{i}$ and $W_{o}$ are the weight matrices, $b_{f},b_{i}$ and $b_{o}$ are the corresponding bias, and σ represents the activation function.

The hidden state is updated using the following formula:

(6) $${\tilde{C}}_{t}=\tanh(W_{C}\cdot [h_{t-1},x_{t}]+b_{C})$$

(7) $$C_{t}=f_{t}*C_{t-1}+i_{t}*{\tilde{C}}_{t}$$

(8) $$h_{t}=o_{t}*\tanh(C_{t})$$

where tanh represents the activation function.

### 3.3. Gated Recurrent Unit

GRU is also a variant of RNN that introduces reset gates and update gates to control the flow of information. Compared to LSTM, GRU has a simpler structure, making it more computationally efficient [43]. The reset gate determines how the new input should be combined with the previous memory, which allows the model to discard or reset part of the previous state, capturing short-term dependencies in the sequence. The update gate decides how much of the current hidden state should be updated, which balances old information with new information, capturing short-term dependencies in the sequence [44]. The mathematical expressions for the reset gate and update gate are as follows:

(9) $$r_{t}=\sigma (W_{r}\cdot [h_{t-1},x_{t}]+b_{r})$$

(10) $$z_{t}=\sigma (W_{z}\cdot [h_{t-1},x_{t}]+b_{z})$$

The hidden state is updated using the following formula:

(11) $${\tilde{h}}_{t}=\tanh(W\cdot [r_{t}*h_{t-1},x_{t}]+b)$$

(12) $$h_{t}=(1-z_{t})*h_{t-1}+z_{t}*{\tilde{h}}_{t}$$

### 3.4. Temporal Convolutional Network

TCN is a neural network architecture specifically designed for processing sequential data with long-range dependencies. Proposed by Bai et al. [45] in 2018, TCN aims to address the issues of vanishing gradients and high computational complexity encountered by traditional RNNs when dealing with long sequences. Unlike traditional RNNs, TCN utilizes one-dimensional causal convolutions, dilated convolutions, and skip connections to effectively capture temporal patterns. It can model long-term dependencies in shorter timeframes and exhibits superior parallel computing capabilities compared to traditional sequential models like RNNs. The TCN model consists of multiple convolutional layers and residual connections, where the output of each convolutional layer is fed into subsequent layers, enabling hierarchical abstraction and feature extraction of sequential data. This multi-scale information extraction capability makes TCN more sensitive to local dependencies in sequential data. TCN also employs residual connections similar to ResNet [46], which effectively alleviate problems such as gradient vanishing and model degradation. Additionally, dilated convolutions expand the receptive field of the convolutional kernel and increase the distance of information propagation, thereby more effectively capturing and modeling long-term dependencies.

### 3.5. Transformer

The transformer is a deep learning model architecture proficient in natural language processing and other sequence-to-sequence tasks. It was introduced by Vaswani et al. [47] in 2017 and consists of four main components: sequence input, encoder, decoder, and sequence output. The transformer architecture introduces a self-attention mechanism, represented by Formula (13), to capture contextual correlations at each position in the input sequence. This mechanism enables the model to consider all positions in the input sequence simultaneously, unlike RNNs or CNNs, which process inputs step by step. The self-attention mechanism allows the model to assign different attention weights to different parts of the input sequence, thereby better capturing semantic relationships. In the transformer, the self-attention mechanism is extended to multiple attention heads, each of which can learn different attention weights to capture different types of relationships [48]. Multi-head attention enables the model to process different information subspaces in parallel. Additionally, transformers are typically composed of multiple identical encoder and decoder layers stacked together, which helps the model learn complex feature representations and semantics [49]. To alleviate the vanishing and exploding gradient problems during training, residual connections and normalization are also employed in the transformer:

(13) $$Attention(Q,K,V)=\mathrm{Softmax}\left(\frac{QK^{T}}{\sqrt{d_{k}}}\right)V$$

where Q,K and V are the query, key, and value vectors computed from the elements of the input sequence, and $d_{k}$ represents the dimension of the key vectors.

### 3.6. Evaluation Criteria of Prediction Performance

RMSE is an error metric used to calculate the error value of the prediction models and to compare the prediction accuracies. RMSE is calculated using the following formula:

(14) $$RMSE=\sqrt{\frac{1}{N}\sum_{i=1}^{N}{\left(y_{i}-{\hat{y}}_{i}\right)}^{2}}$$

where *N* is the total number of samples, and $y_{i}$ and ${\hat{y}}_{i}$ are the true and predicted values of the *i*th sample, respectively.

## 4. Results and Discussion

Despite the sampling frequency of the sensor being 0.4 Hz during the durability testing, the SOH of the fuel cell did not exhibit significant changes over short periods. This implies that it is not highly sensitive to temporal variations. Therefore, to investigate the impact of resampling frequency on the predictive performance of models for the SOH of PEMFCs and provide guidance for practical predictive tasks, we resampled the durability test data at intervals of 20 min, 40 min, and 60 min. In addition to the sampling frequency, the quantity of training samples in the training process is also a crucial factor affecting the predictive performance of the model. We conducted independent experiments using 70%, 50%, and 30% of the total sample size for training. Detailed results and in-depth analysis are presented in this section.

### 4.1. Data Analysis

**Figure 4:**
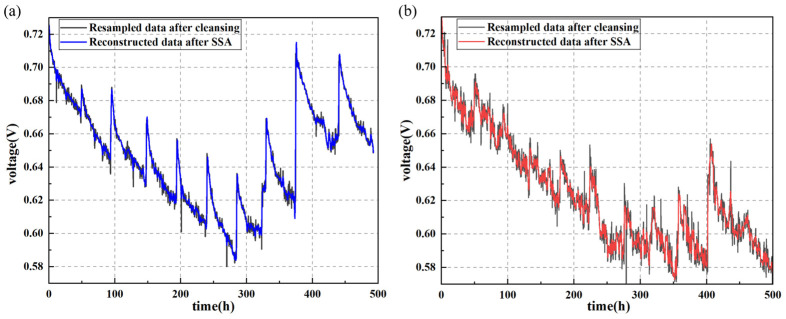
Resampled data after cleansing at intervals of 20 min and reconstructed data after SSA for voltage–time curves. (**a**) PEMFC without cracks; (**b**) PEMFC with uniform cracks.

During the durability test, the output voltage of the fuel cell gradually decreased, but the decline was neither smooth nor constant. Each sampling interval contained data points with the same current density. Therefore, the average output voltage at the same current density around each sampling time point was calculated as the experimental value for that data point. Additionally, anomalies can occur due to noise from the sensor equipment. Thus, data cleaning is performed to remove missing and anomalous data elements collected during the experiment. To further improve data quality, the experimental data were smoothed using SSA. The comparison of the experimental data before and after the treatment is shown in Figure 4.

**Figure 5:**
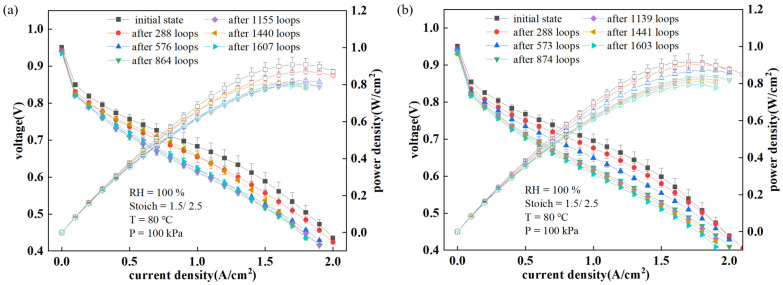
Polarization curves and power density curves of the PEMFCs. (**a**) PEMFC without cracks; (**b**) PEMFC with uniform cracks.

When electrochemical measurements were conducted after the fuel cell stopped operating, the distribution of gas and liquid inside the fuel cell changed [50], causing a temporary and reversible increase in fuel cell performance when the durability experiment resumed. This phenomenon, which can also occur in actual operation, is retained in the dataset prior to prediction work. From Figure 4, it is also evident that the average output voltage of the fuel cell with uniform cracks is lower. Moreover, Figure 5 shows the polarization curves and power density variations of the two PEMFCs during the durability test. In the initial state, the PEMFC with uniform cracks is more efficient, which can be attributed to the fact that cracks in the catalyst layer can initially enhance the transport of reactants and byproducts, reducing mass transport resistance and improving performance. However, these cracks also create sites for accelerated degradation processes, such as increased susceptibility to corrosion and mechanical wear. Over the course of cycles, these degradation processes become more pronounced, leading to a faster decline in performance. The performance degradation caused by cracks may be due to following. First, the cracks alter the microstructure of the catalyst layer, reducing the number of active catalysts and affecting the efficiency of gas diffusion to the catalyst active sites. This reduction in active sites and the efficiency of reactant delivery directly impacts the electrochemical reactions, thereby lowering the overall performance of the fuel cell. Second, the presence of cracks may lead to uneven current density distribution within the fuel cell. Localized areas may experience higher current densities, which can accelerate the aging and failure of these regions. This uneven distribution of current density is detrimental to the longevity and reliability of the fuel cell, as it causes certain areas to deteriorate faster than others. Moreover, during the reaction process, cracks may propagate further, leading to additional structural degradation. This propagation can affect the integrity of other components within the fuel cell, further impairing its performance. The expansion of cracks not only disrupts the physical structure but also potentially alters the electrochemical environment, making the conditions less favorable for optimal operation.

### 4.2. Data Resampling Intervals

In the fuel cell durability prediction work, we set up experiments with different sampling intervals to generate datasets. The specific procedure involved sampling data points at intervals of 20 min, 40 min, and 60 min and then dividing the data collected at each sampling interval into training, validation, and testing sets. In this setup, 30% of the sampled data were allocated to the training set for model training, 10% to the validation set for validation, and the remaining 60% were assigned to the testing set for evaluating model performance.

**Figure 6:**
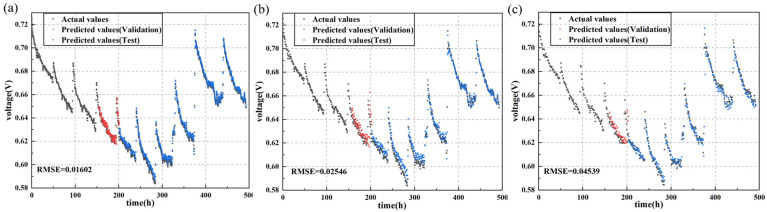
Actual vs. prediction values for output voltage of the PEMFC without cracks predicted with LSTM using data resampling intervals of (**a**) 20 min, (**b**) 40 min, and (**c**) 60 min.

**Figure 7:**
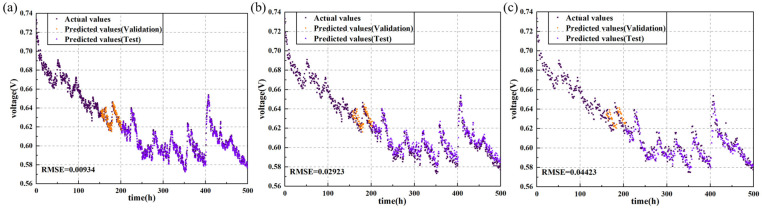
Actual vs. prediction values for output voltage of the PEMFC with uniform cracks predicted with LSTM using data resampling intervals of (**a**) 20 min, (**b**) 40 min, and (**c**) 60 min.

As shown in Figure 6 and Figure 7 and Table 2, datasets constructed with shorter sampling intervals contributed to improved predictive performance of the models. Specifically, models trained on datasets with 20 min intervals exhibited smaller RMSE in their predictions. For the LSTM model, reducing the sampling interval from 60 min to 40 min resulted in a 43.9% decrease in RMSE, and further reducing the interval from 40 min to 20 min decreased the RMSE by 37%. When the sampling interval was 20 min, the model achieved an RMSE of 0.01602 on the testing set, and it can be observed from Figure 3 that the predicted values on the testing set closely matched the experimental values, indicating successful completion of the prediction task.

Furthermore, as indicated in Table 2, other models proficient in time-series prediction, such as GRU and TCN, exhibited similar trends of improved predictive performance with shorter sampling intervals. When the sampling interval was 20 min, these models achieved the lowest RMSE. This suggests that shorter sampling intervals significantly enhance the predictive accuracy for these models. The only exception was the transformer model, which achieved a lower RMSE with a 40 min sampling interval, possibly due to the unique architecture and approach to handling time-series data in the transformer model.

Additionally, regarding the two sets of durability data provided, the LSTM model demonstrated the best predictive performance across the three sampling intervals. For durability data of fuel cells without cracks, when the sampling interval was set to 20 min, the RMSE was reduced by 35.8%, 5.15%, and 69.4% compared to GRU, TCN, and transformer, respectively. For durability data of fuel cells with uniform cracks, when the sampling interval was set to 20 min, the RMSE was reduced by 27%, 60%, and 71.5% compared to GRU, TCN, and transformer, respectively.

The reason behind these observations is that shorter sampling intervals allow for the generation of more data points, capturing transient changes and detailed information more effectively. These high-resolution data better describe the dynamic behavior of fuel cells. During testing, models leveraging this detailed information can predict voltage changes in fuel cells more accurately. Data sampled at shorter intervals encompass more subtle variations in the state of the fuel cell, enabling the models to identify and learn more patterns and features. In contrast, longer sampling intervals may overlook these details, resulting in weaker responsiveness to rapid changes.

Therefore, in terms of prediction accuracy, models trained on datasets with shorter sampling intervals outperform those trained on datasets with longer intervals. Shorter sampling intervals, by capturing more transient changes and detailed information, enhance the models’ ability to describe the dynamic behavior of fuel cells and achieve more accurate voltage predictions during testing.

### 4.3. Training Dataset Size

To further investigate the impact of training set proportions on model prediction performance, we chose to set the sampling interval to 20 min to provide the models with sufficient and timely data. We then set the training set proportions to 70%, 50%, and 30%, with 10% of the data used as a validation set, and the remaining data used to evaluate the model’s prediction performance.

**Figure 8:**
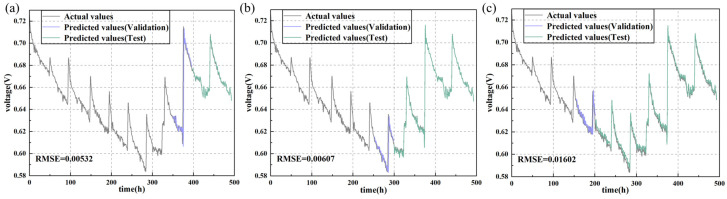
Actual vs. predicted values for output voltage of the PEMFC without cracks predicted by LSTM with different training proportions: (**a**) 70%; (**b**) 50%; (**c**) 30%.

**Figure 9:**
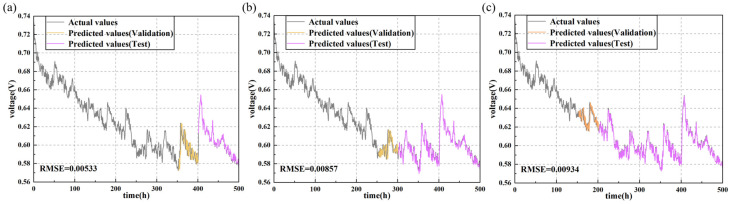
Actual vs. predicted values for output voltage of the PEMFC with uniform cracks predicted by LSTM with different training proportions: (**a**) 70%; (**b**) 50%; (**c**) 30%.

As depicted in Figure 8 and Figure 9, increasing the training set proportion significantly improves the accuracy of the prediction models. For instance, in the LSTM model, on the durability test set of fuel cells without cracks, the RMSE decreases as the training set proportion increases. Specifically, when the training set proportions are 70%, 50%, and 30%, the RMSE values are 0.00532, 0.00607, and 0.01602, respectively. Similarly, on the durability test set of fuel cells with uniform cracks, the LSTM model exhibits a similar trend. When the training set proportion increases from 30% to 50%, the RMSE decreases by 37.8%, and further decreases by 8.2% when the training set proportion is increased to 70%.

Table 3 shows the prediction performance of various deep learning models under different training set proportions. In both durability datasets, increasing the training set proportion provides the models with more information, thereby significantly improving their prediction accuracy. For example, the GRU model’s prediction performance improves by 78% and 16.2% on the two datasets when the training set proportion increases from 30% to 70%. The TCN model’s prediction performance improves by 48.6% and 60.6% on the two datasets, while the transformer model’s prediction performance improves by 57.5% and 35.8%. Despite these improvements, the LSTM model consistently shows the best performance, whereas the transformer model’s performance is relatively poorer.

Additionally, it is evident that the presence of cracks leads to more frequent and intense voltage fluctuations, which also affect the prediction models’ performance. Specifically, cracks cause changes in the microstructure of the fuel cell, increasing the instability of electrochemical reactions, which leads to uncertainty in voltage output. This unstable voltage fluctuation makes it more challenging for models to accurately capture the true dynamic behavior of the fuel cells, thereby impacting prediction accuracy. In contrast, crack-free fuel cell systems are more stable with relatively smooth voltage changes, allowing models to learn and predict their behavior patterns more effectively. Therefore, under crack-free conditions, prediction errors are smaller and performance is superior. This trend is evident in the performance evaluations of all models, including LSTM, GRU, TCN, and transformer.

## 5. Conclusions

In this study, we extensively investigated the methods and effectiveness of accurately estimating the SOH of PEMFCs under dynamic operating conditions using deep learning models. Various types of deep learning models, including LSTM, GRU, TCN, and transformer, were employed, and dynamic durability tests were conducted on both crack-free and uniformly cracked PEMFCs. By varying the sampling intervals and training set proportions, we evaluated the impact of these factors on the predictive performance of the models.

The results indicated that moderately increasing the training set proportion and reducing the sampling interval significantly improved the predictive accuracy of deep learning models. In particular, under crack-free conditions, the LSTM model exhibited outstanding performance, with a substantial decrease in RMSE as the training set proportion increased. For instance, with a sampling interval of 20 min and a training set proportion of 70%, the LSTM model achieved an RMSE of only 0.00532. However, the presence of cracks posed challenges to the model performance, as cracks led to more frequent and intense voltage fluctuations, making it difficult for the models to capture the degradation behavior of PEMFCs.

In summary, this study compared multiple deep learning models, emphasizing the effectiveness of deep learning models in improving the accuracy of estimating the SOH of PEMFCs and emphasizing the importance of optimizing sampling and training strategies to further improve prediction accuracy. In addition, it provides insights into the impact of cracks on fuel cell performance and SOH estimation. Furthermore, this study is based on dynamic durability test data, helping to promote research on and use of SOH estimation strategies for PEMFCs in various practical applications, and is of great significance for developing more reliable and efficient PEMFCs and promoting sustainable energy technologies. In future work, we aim to further enhance the models to reduce the error between predicted and actual values and conduct more research on the effects of cracks on fuel cell performance.

## Figures and Tables

**Table 1:** The technical parameters of the fuel cells.

Technical Parameters	Value
Activated area	5 × 5 cm^2^
Thickness of membrane	12 μm
Thickness of GDL	220 μm
Thickness of CL	15 μm
Radius of Pt particle	5 nm
Pt loading of the anode	0.05 mg·cm^−2^
Pt loading of the cathode	0.3 mg·cm^−2^

**Table 2:** Model accuracy with training proportion of 30% using data resampling intervals of 20 min, 40 min, and 60 min.

		RMSE (20 min)	RMSE (40 min)	RMSE (60 min)
PEMFC without cracks	LSTM	0.01602	0.02546	0.04539
	GRU	0.02495	0.02574	0.05289
	TCN	0.01689	0.03968	0.04866
	Transformer	0.05242	0.05017	0.08404
PEMFC with uniform cracks	LSTM	0.00934	0.02923	0.04423
	GRU	0.0128	0.03297	0.03911
	TCN	0.0234	0.03908	0.05153
	Transformer	0.03273	0.03424	0.05219

**Table 3:** Model accuracy with data resampling intervals of 20 min using different training proportions of 70%, 50%, and 30%.

		RMSE (70%)	RMSE (50%)	RMSE (30%)
PEMFC without cracks	LSTM	0.00532	0.00607	0.01602
	GRU	0.00546	0.00831	0.02495
	TCN	0.00867	0.01516	0.01689
	Transformer	0.0223	0.03349	0.05242
PEMFC with uniform cracks	LSTM	0.00533	0.00857	0.00934
	GRU	0.01073	0.01242	0.0128
	TCN	0.00922	0.01604	0.02341
	Transformer	0.02101	0.03053	0.03273

## Data Availability

The data presented in this study are available on request from the corresponding author.
